# What Are Animal Rights For?

**DOI:** 10.1017/awf.2024.33

**Published:** 2024-09-16

**Authors:** Eva Bernet Kempers

**Affiliations:** Postdoctoral Researcher Animal Law at the University of Antwerp

The book *What Are Animal Rights For?* offers a concise yet compelling exploration of animal rights that transcends the traditional philosophical discourse by delving into the practical implications of the ethical framework of animal rights for contemporary society.

The book starts by introducing the notion of ‘sentience’ which is taken as the main characteristic on which animal rights are grounded. In the second chapter, the author then meticulously traces the historical trajectory of animal rights, weaving together seminal works by renowned philosophers like Regan and Singer with often-overlooked perspectives from non-Western thinkers such as Al-Ma’arri. Hence, the more known schools of social contract theory, virtue ethics, Kantian ethics, and utilitarianism are covered next to thinkers that are less widely known. By spanning the intellectual history from ancient Greece to the modern era, the book elucidates the profound impact of early ideas on the evolution of the animal rights movement. After that, it succinctly sets out the framework of rights and interests in Chapter 3, in order to provide the reader with a foundation for the ensuing discussions. Here, the main aim is to demonstrate how animal rights theory responds to the claim that only human beings can have rights. Interestingly, the author speaks of the argument from ‘awkward cases’ instead of the traditional ‘marginal cases’ in this regard – a move that is commendable, as the label of this argument has been subject of critique in recent years. In Chapter 4, the author uses four case studies: animal agriculture, companion animals, zoos, and animals in experimentation, to further illustrate what animal rights would mean in practice. Here, some pictures are used to give faces to the animals involved in these practices. Then, it introduces some emerging issues, such as the question of invertebrate animal rights and the citizen-theory as applied to animals in Chapter 5. The collection of these three issues (invertebrates, wild animals, and animal citizens) seems somewhat arbitrary, but forms a welcome extension of the more commonly used categorisation of Chapter 4. In Chapter 6, the discussion culminates in a more practical plea to turn prejudice into compassion for animals, assessing the role of emotions in the animal rights movement. The conclusion adds that, apart from rights, a cultural shift is necessary in order to truly ensure respect for animals and foster relationships of trust between all living beings.

The book is an entertaining read, and for someone like the author of this book review, who has been working in animal law for over five years, it still inspires some new thoughts and ideas. In particular the section on invertebrate animals is a crucial and up-to-date addition to the traditional narrative of animal rights. Overall, one of the book’s greatest strengths lies in its accessibility to a broad audience. By eschewing overly technical language and focusing on real-world situations, it serves as an ideal entry-point for readers new to the subject of animal rights. The focus is on the meaning of animal rights in practice, rather than the academic field of ‘animal rights theory’. The author really seeks to inspire us to think about what our society would look like if animal rights were to be respected. A further strength of the book is its use of case studies, which make the theoretical discussions more tangible. In some ways, the approach of the book reminds us of Nussbaum’s *Justice for Animals* – in a similar way, the author connects theory to practical contemporary situations – yet in a less detailed and theoretical and thus more accessible manner. Lastly, the relevance of emotions as discussed in Chapter 6 clearly distinguishes the book from the more rationalistic accounts of the late 20^th^ century.

Nevertheless, the accessibility of the book at times comes at the cost of academic rigour, as the author occasionally glosses over complex theoretical debates and fails to fully articulate the underlying framework of their argument. For instance, when going over the idea of ‘animals as citizens’, there is no discussion of the theoretical framework of *Zoopolis*, which is clearly the basis for this idea. Furthermore, the case studies are used more as illustrations than as a way to analyse how the inconsistencies of the animal welfarist paradigm are manifested in practice. Lastly, due to the lack of attention for how and at which level animal rights would be legally codified (e.g. in animal rights laws, or in international conventions), the legally oriented proponents of animal rights are left with a bit of a hunger, as the book seems to be mainly concerned with rights as a political discursive tool, rather than rights in a legal sense. Hence, the book should mainly be regarded a good first step into the topic of animal rights, for those who do not need to know the more thorough theoretical underpinnings or legal implications of the subject.

Despite these limitations, *What Are Animal Rights For?* serves as a thought-provoking exploration of an increasingly urgent ethical issue. Its engaging prose and practical focus make it a valuable resource for both general readers and students alike, extending the conversation beyond the confines of academia and into the broader societal discourse on animal welfare and rights. The author takes the reader by the hand, demonstrating the relevance of animal rights theory for our current societies, making a bridge between theory and practice. It is an entertaining read for the broader public, as well as students interested in the topic, and I warmly recommend it to anyone open to being convinced that a society in which animal rights are being respected, is in fact possible.

